# Damage Detection in Flexible Plates through Reduced-Order Modeling and Hybrid Particle-Kalman Filtering

**DOI:** 10.3390/s16010002

**Published:** 2015-12-22

**Authors:** Giovanni Capellari, Saeed Eftekhar Azam, Stefano Mariani

**Affiliations:** 1 Politecnico di Milano, Dipartimento di Ingegneria Civile e Ambientale, Piazza L. da Vinci 32, 20133 Milano, Italy; giovanni.capellari@polimi.it (G.C.); stefano.mariani@polimi.it (S.M.); 2 University of Thessaly, Department of Mechanical Engineering, Leoforos Athinon, Pedion Areos, 38334 Volos, Greece

**Keywords:** structural health monitoring, reduced-order modeling, proper orthogonal decomposition, particle-Kalman filtering, inertial sensors

## Abstract

Health monitoring of lightweight structures, like thin flexible plates, is of interest in several engineering fields. In this paper, a recursive Bayesian procedure is proposed to monitor the health of such structures through data collected by a network of optimally placed inertial sensors. As a main drawback of standard monitoring procedures is linked to the computational costs, two remedies are jointly considered: first, an order-reduction of the numerical model used to track the structural dynamics, enforced with proper orthogonal decomposition; and, second, an improved particle filter, which features an extended Kalman updating of each evolving particle before the resampling stage. The former remedy can reduce the number of effective degrees-of-freedom of the structural model to a few only (depending on the excitation), whereas the latter one allows to track the evolution of damage and to locate it thanks to an intricate formulation. To assess the effectiveness of the proposed procedure, the case of a plate subject to bending is investigated; it is shown that, when the procedure is appropriately fed by measurements, damage is efficiently and accurately estimated.

## 1. Introduction

The health monitoring of aging structures and infrastructures [1,2,3,4] is nowadays becoming more and more important, and can exploit devices and methodologies developed within the field of embedded or inclusive smart technologies [5,6,7]. The envisioned structural health monitoring (SHM) systems have to sense in real-time the changing environment, so as to send out early warnings if dangerous situations are approached. Such feature would also be appealing if structures have to be monitored in regions where environmental and geological risks are of concern [8].

To provide some data highlighting the timeliness of smart SHM technologies, it is worth noting that a high percentage of civil structures and infrastructures in the developed and industrialized nations was built in the first half of the twentieth century: over 50% of the bridges in the USA were built prior to 1940 [9]; furthermore, over 42% of all the aforementioned bridges are structurally deficient, as reported in [10]. In Canada, over 40% of the bridges were built earlier than 1970, and a majority of them demands prompt rehabilitation, strengthening or replacement [11].

The aim of this paper is the development of an online damage identification method, or SHM strategy based on recursive Bayesian filters [12]. Such filters have been already successfully applied to the (grey-box) identification of shear-type buildings: in [13], unscented Kalman and particle filters were adopted for the online parametric identification of nonlinear hysteretic models with uncertainties; in [14], an efficient extended Kalman-particle filter scheme was adopted for the online identification of a structural model featuring a softening inter-story behavior. Recently, the attention has also been drawn to input-state estimation so that the obtained structural response can be used for the fatigue damage assessment, via vibration measurements, of structures subject to cyclic loadings [15,16].

The focus of the present study is on the design of a SHM strategy featuring fast, robust and unbiased estimations of damage indexes, obtained thanks to partial observations of the structural response. Within this frame, the aforementioned local damage indexes quantify the reduction of the stiffness properties of the structure. A similar approach can be adopted to also estimate the local reduction of the materials strength properties, although this topic is out of the scope of the current work and will be investigated in future activities. We therefore assume that the structure behaves piece-wise linearly, within any time window between subsequent observations of the structural response; accordingly, only the time-varying elastic properties of the system need to be identified. The goal of fast and accurate monitoring of these systems is here attained by introducing an effective model order reduction technique, to guarantee low computational costs, and by optimizing the deployment of a limited number of (inertial) sensors, collecting the observations of the system response.

The model order reduction is achieved through a Galerkin projection of the original full structural model onto a sub-space spanned by the so-called proper orthogonal modes (POMs), computed via proper orthogonal decomposition (POD) in its snapshot version [17,18,19]. The simultaneous, dual estimation of the reduced-order structural state and of the mentioned damage indexes is obtained with a particle filter, enhanced through the use of an extended Kalman filter before the resampling stage [14,19]. The analysis of such dual estimation procedure, featuring an augmented state vector that gathers both the state of the system and the parameters to be tuned, can be traced back to [20] and, somehow, to the seminal paper [21]. In recent years, nonlinear dual estimation problems related to structural dynamics have been tackled with the use of the extended Kalman filter (see [22,23] among others), of the unscented Kalman filter [24], of particle filters [25,26], and of hybrid particle-Kalman filters [19,27]. Hence, in this work we do not focus on the development of a new filtering technique but we instead show how a hybrid particle-Kalman filter can be adopted to identify on-the-fly the time-dependent properties of a reduced-order model of the structural system.

It is known that, as number of parameters to be identified increases, the accuracy of identification tends to decrease: this issue can be linked to the so-called curse of dimensionality. When dealing with the identification of structural systems featuring a large number of degrees-of-freedom (DOFs), methods like component mode synthesis were developed for reducing the number of unknown system parameters in procedures for model updating; with this approach, modal properties are identified offline. In this study, the goal is the online and real-time estimation of the damage parameters of the system, so the POMs are directly obtained from the response of the system in the initial training phase. As the inception and growth of damage modify the structural properties and, thereby, the relevant response to the external actions, the sub-space spanned by the POMs needs to be continuously updated during the filtering process; such update is here obtained thanks to a further Kalman filter. The resulting intricate formulation allows tracking the time evolution of the damage parameters as well as of the state of the partially observed system.

To assess the capability of the proposed SHM procedure, a thin plate subject to bending-dominated deformations and (possibly) time evolving damage is considered. It is shown that the filter is able to identify the spatial distribution of damage even if an extremely low number of POMs (on the order of 1 to 4) is adopted. This strongly reduced-order of the model handled by the filter is not strictly related to the kind of excitation considered; it takes instead advantage of the filter adopted to track the evolution of the POMs, which beneficially affects the overall accuracy of the procedure. Concerning observations, it has been assumed that a network of inertial sensors is surface-mounted on the plate, according to what proposed in [28,29]. To investigate the computational advantage of the proposed method, the computational complexity and the CPU time have been considered: it is demonstrated that the proposed method can speed-up the analyses up to hundreds of times in comparison to the full finite element model.

Methods similar to the one adopted herein for dual estimation and sub-space update, were recently developed and successfully applied to shear-type buildings in [18,19]. The current study represents an evolution of previous works, as it proves to be able to locate and estimate a structural damage via a few vibration measurements only.

The remainder of this paper is organized as follows. In Section 2 the proposed SHM strategy is described: first, the model order reduction approach is detailed; next, the intricate filtering strategy, developed to simultaneously track the damage evolution and the response of the structure in the reduced-order space, is reported. In Section 3, results are shown for the SHM of a thin square plate displaying a reduction of its stiffness properties. Some closing remarks and suggestions for future developments are gathered in Section 4. Finally, Appendix A collects some major algorithmic details of the proposed procedure.

## 2. Methodology

As already discussed, the health monitoring of real-life structures with complex geometry and/or boundary and loading conditions can become extremely time consuming, as an accurate model to interpret the collected measurements has to be provided.

In order to send out warnings as soon as dangerous conditions are approached, the SHM procedure cannot be run offline; accordingly, a reduced-order model of the system has to be set with a high level of fidelity; this issue is here tackled through POD. Since reduced-order modeling introduces additional uncertainties into the structural model, the Bayesian procedure used to provide the estimations of damage amount (if any) and location can become inaccurate. Such problem is next handled through a fully automated filtering methodology able by itself to enhance the accuracy of the reduced-order model, adapt it to possible structural changes and simultaneously estimate the local damage.

### 2.1. Model Order Reduction

The dynamic behavior of any space-discretized (e.g., through finite elements) mechanical system can be described through the following vector-valued equation of motion [19]:

(1) $$M\ddot{u}\left(t\right)+D\dot{u}\left(t\right)+Ku\left(t\right)=F\left(t\right)$$

where t is time; $u\in {\mathbb{R}}^{n}$ is the vector that collects the kinematic quantities describing the structural response, either displacements or rotations for the considered plates; as a superposed dot stands for time derivative, $\dot{u}$ and $\ddot{u}$, respectively, gather the relevant velocities and accelerations; M is the mass matrix; D is the viscous damping matrix; K is the stiffness matrix; and F is the vector of the external forces.

The model order reduction technique is set to decrease the dimension of the vectors gathering the kinematic quantities and their time derivatives and, therefore, of the matrices in Equation (1). The reduction is obtained via a projection of system dynamics onto a low order sub-space, leading to:

(2) $$ℳ\ddot{\alpha }\left(t\right)+D\dot{\alpha }\left(t\right)+K\alpha \left(t\right)=ℱ\left(t\right)$$

where $\alpha \in {\mathbb{R}}^{l}$, with l≪n, and [18,19]:

(3) $$\begin{array}{c} ℳ=\Phi_{l}^{T}M\Phi_{l}\\ D=\Phi_{l}^{T}D\Phi_{l}\\ K=\Phi_{l}^{T}K\Phi_{l}\\ ℱ=\Phi_{l}^{T}F\;\;\;\;\; \end{array}$$

where, above, $\Phi_{l}\in {\mathbb{R}}^{n\times l}$ is a matrix wherein POMs are arranged according to:

$$\Phi_{l}=\left[\begin{array}{ccc} \Phi^{1} & \cdots & \Phi^{l} \end{array}\right]$$

This projection matrix allows linking the kinematic variable vectors in Equations (1) and (2) through $u\left(t\right)≅\Phi_{l}\alpha \left(t\right)$, and gathers the required number l of POMs to attain an *a-priori* defined degree of (energy-like) accuracy. Details relevant to this topic can be found in [18,19,30,31] and are not further discussed here. POMs are obtained through a principal component analysis of a matrix built around the so-called structural snapshots, which provide the evolution in time of the system response to the external loads (see [17] and [32]). The snapshots are collected in the initial training stage of the reduced-order modeling procedure. The computational burden of this stage is basically linked to the number of snapshots that guarantee the convergence towards a steady-state solution $\Phi_{l}$; adaptive procedures are available to reduce this to a minimum, see e.g., [32]. As already reported in [18], for systems like shear buildings subject to earthquake excitations, the duration of the training stage can be simply based on the fundamental period of vibration of the structure, and a collection of around 30−50 snapshots proves sufficient to approach the mentioned steady-state.

POD has been devised for linear systems; to cope with an evolving damage state, leading to a time-dependent stiffness matrix K, the POD is here coupled with a Kalman filter that continuously adapts the sub-space onto which the model evolution is projected. Such simultaneous use of a filter has been devised for systems whose nonlinearity slowly evolves in time; if abrupt changes of the system properties occur, the proposed procedure requires some time for the reduced-order model to adapt to the new structural health, and so some delay in the tracked damage conditions can emerge.

### 2.2. Damage Detection and Localization

To estimate the possible local damage, the SHM procedure is driven by the reduced-order model only. The problem is framed as a dual estimation one, within which both the dynamic evolution of the structure and its local integrity are retained in a state vector that reads, at time $t_{k}$:

(4) $$x_{k}=\left\{\begin{array}{c} \begin{array}{c} \alpha_{k}\\ {\dot{\alpha }}_{k} \end{array}\\ \begin{array}{c} {\ddot{\alpha }}_{k}\\ d_{k} \end{array} \end{array}\right\}$$

where vector $\alpha_{k}$ collects the current values of the generalized coordinates associated with the reduced-order model; vector $d_{k}$ collects instead the local damage indexes $d_{i,k}$ (for $i=1,\ldots ,N_{p}$) of all the $N_{p}$ zones in which the structure is conceptually decomposed. Hence, it turns out that $x_{k}\in {\mathbb{R}}^{3l+N_{p}}$. As reported in Equation (4), not only the generalized coordinates $\alpha_{k}$ but also their time derivatives ${\dot{\alpha }}_{k}$ and ${\ddot{\alpha }}_{k}$ are retained in $x_{k}$. Such increase of the number of the state components has been already targeted in [24,33,34] as a necessary condition to track the nonlinear evolution of the statistics of the system within a stochastic framework. In fact, in the presence of dissipative phenomena like the considered degradation of the mechanical properties of the structure, activation conditions are typically adopted to model the evolution of internal state variables (in our case, of the damage indexes), and so complementarity conditions arise. The Jacobian of the state evolution equations, that will be shown to have a role in the filtering procedure, is linked to such activation conditions; the evolution of the state variables thus becomes path-dependent, and requires all the kinematic variables to be gathered in the augmented state vector $x_{k}$.

Considering all the equations governing the evolution of system and damage indexes in a stochastic environment, the dynamics of the augmented state vector in the generic time interval $\left[t_{k-1}\;t_{k}\right]$ reads:

(5) $$x_{k}=f_{k}\left(x_{k-1}\right)+\;w_{k}$$

where $w_{k}$ is a zero mean, additive white Gaussian noise with covariance W, that represents the uncertainties related to the mathematical/numerical model of the system and, further to any standard full-order formulation, the errors introduced by the reduced-order modeling; the nonlinear operator $f_{k}$ depends on the adopted time integration algorithm and on the mechanical characteristics of the structure, *i.e.*, on the matrices ℳ,D and K showing up in the reduced-order model. A thorough discussion on this topic is provided in Appendix A, see also [14].

To model the structural effects of damage, we now follow a rather standard approach in damage mechanics, see e.g., [34]: the mass and damping matrices are not affected by the damage state; the stiffness matrix is instead assumed to scale proportionally to the damage. To account for multiple damage scenarios with different patterns, the reduced-order stiffness matrix K is represented as a linear combination of $N_{p}$ sub-matrices, each one relevant to a region featuring a uniform damage:

(6) $$K_{k}=\overset{N_{p}}{\underset{i=1}{\sum }}\left(1-d_{i,k}\right)K_{und}^{i}$$

where the index k is now also adopted for K to denote that its value is damage-dependent, and therefore time-dependent. The summation in Equation (6) has to be correctly interpreted as the assemblage [35] of the undamaged stiffness contributions $K_{und}^{i}$, all scaled by the corresponding values of the damage indexes. Equation (6) shows that damage indexes are dimensionless variables. Here, we do not aim to model the evolution of damage induced by the current loading; we therefore assume that indexes $d_{i,k}$ are material parameters, which might change in time (according to thermodynamic requirements, they can only monotonically increase in time in the range [0–1)) and are homogeneous inside each region (so, independent of the adopted space discretization inside the region itself).

The structural response thus becomes nonlinear whenever damage grows. The linear structural dynamics described by Equation (1) or (2) does not provide any means to explicitly model such evolving state of the system; by assuming in the filtering procedure that K can evolve in time in a step-wise fashion, such issue gets negligible if the time scales corresponding to the damage evolution and to the monitored structural response are well separated.

To estimate the state of the system and the hidden (*i.e.*, not explicitly observable) damage parameters, observations of the system look necessary. Allowing for the formulated reduced-order model of the structure, the stochastic measurement equation reads

(7) $$y_{k}=HL_{k}x_{k}+v_{k}$$

and provides a link of the $N_{obs}$ observations collected in the vector $y_{k}$ with the reduced-order state vector $x_{k}$. Here, H is an appropriate Boolean matrix that allows to pick up the components of u, $\dot{u}$ and $\ddot{u}$ actually observed. Since the type of sensors and their positions are not supposed to change in time, H turns out to be time-independent. In Equation (7), $v_{k}$ is a zero mean, additive white Gaussian noise featuring covariance V, that represents the uncertainties due to measurements, typically related to the accuracy of the sensors deployed over the structure. As observations in $y_{k}$ are defined in the full-order space while $x_{k}$ lies in the reduced sub-space, the transformation matrix $L_{k}$ is required for the state vector to be recovered in the original space. According to the definition (4) of $x_{k}$, we therefore have:

(8) $$L_{k}=\left[\begin{array}{cccc} \Phi_{l,k} & & & \\ & \Phi_{l,k} & & \\ & & \Phi_{l,k} & \\ & & & 0 \end{array}\right]$$

where $\Phi_{l,k}$ is the projection matrix estimated at time $t_{k}$, and all the off-diagonal matrices not reported are null. Matrix $L_{k}$ obviously features null diagonal block corresponding to the vector $d_{k}$ of the non-observable damage variables, as they do not have a role in the sub-space projection.

Damage evolution in the structure depends on the loading conditions; hence, the evolution in time of the POMs linked to the changes of the structural health, cannot be *a-priori* assigned. In our approach, we assume that damage evolution is smooth and slow enough, so that POMs can be considered constant within each time step. Since in a Kalman filtering implementation state variables are all gathered in vector form, POMs of the reduced model are rearranged now inside vector $\varphi_{l,k}\in {\mathbb{R}}^{nl}$ according to:

$$\varphi_{l,k}=\left\{\begin{array}{c} \Phi_{k}^{1}\\ \vdots \\ \Phi_{k}^{l} \end{array}\right\}$$

Within a stochastic frame, we can therefore model the evolution of the projection sub-space as:

(9) $$\varphi_{l,k}=\varphi_{l,k-1}+w_{k}^{ss}$$

where $w_{k}^{ss}$ is a fictitious zero mean Gaussian noise, whose covariance $W^{ss}$ is to be tuned in order to guarantee stability and accuracy of the estimations. The measurement equation relevant to the sub-space tracking reads:

(10) $$y_{k}=H_{k}^{ss}\;\varphi_{l,k}+v_{k}$$

where $H_{k}^{ss}$ is an appropriate matrix that links the observations $y_{k}$ and the sub-space vector $\varphi_{l,k}$. It must be noted that Equations (7) and (10) do represent the same link between observables $y_{k}$, state variables $x_{k}$ and POMs $\Phi_{k}^{i}$, with i=1,…,l; they are only written in somehow different forms to explicitly state what is identified in the two stages of the proposed procedure, namely $x_{k}$ first and$\;\varphi_{l,k}$ next.

Sensor inaccuracies can potentially affect sub-space and damage estimates. As far as the sub-space estimation is concerned, it should be noted that the POMs are the set of basis vectors which optimally capture the maximum variations in the structural response. Therefore, a constant unexpected offset in the sensor measurement is not supposed to alter the estimates of POMs; conversely, in case of a time varying offset, POMs may be affected. To alleviate issues raised by an inaccurate calibration of noises, some methodologies were proposed for their online and real-time, yet optimal estimates through Kalman-type filters, see e.g., [36,37]. While the focus of these works was on developing Bayesian probabilistic methods for noise covariance estimation, it was shown in [37] that they could be generalized to also account for a time variability of the noise mean value.

To cope with all the details reported here above, the adopted recursive Bayesian method has been developed starting from what proposed in [19]: a particle filter is exploited first to estimate the state vector $x_{k}$, given a certain set of data $y_{k}$ acquired from the network of sensors deployed over the structure; next a Kalman filter is adopted to update the POMs, still on the basis of observations $y_{k}$. The latter stage is obviously necessary whenever the damage state changes, and so a different structural response arises; thanks to the final update stage of the Kalman filter, biases in the observables can be exploited to achieve model updating. Both filtering procedures deliver the estimate of damage or the model updating via two stages: first, a prediction is achieved through the evolution or state equations; second, the prediction is updated by taking advantage of observations. Prediction is usually pursued through Chapman-Kolmogorov integrals, whereas the update through the Bayes rule of conditional probability. A closed form solution to the aforementioned procedure can be found only for linear systems with white Gaussian uncertainties. In case of a nonlinear state-space model featuring weak nonlinearities, the solution is obtained by linearizing the state-space equations; if instead the probability distribution of the state variable is non-Gaussian or if severe nonlinearities prevail, the Chapman-Kolmogorov integrals can be computed with numerical quadrature rules. A well-known method to deal with general nonlinear, non-Gaussian systems is the adopted particle filter [38], which is based on a Monte Carlo sampling performed according to the posterior probability distribution of the state variables. As such distribution is not available while sampling, a major aim of the filtering method is to approximate it. Alternatively, sequential importance sampling [38] usually makes recourse, in the absence of the optimal distribution, to the prior probability density function of the state variables. Hence, with this latter approach samples drawn do not contain information brought by the latest observations.

According to what discussed, particle filtering has been preferred to other methods, e.g., the extended or unscented Kalman filtering [14,19,33,34], in order to better match the real non-Gaussian statistics of the state of the nonlinearly evolving system. As this approach would require a large set of particles to get an accurate estimation of damage, an extended Kalman filter is adopted to push the samples towards zones of higher probability and therefore reduce the overall number of particles to be adopted [19].

To perform the dual estimation, a linearization of the evolution Equation (5) is required. In Algorithm 1, $F_{k}$ denotes the Jacobian of function $f_{k}$ computed with the currently available estimation $x_{k-1}$ of the state of the system; further details are provided in Appendix A. The whole procedure, as detailed in Algorithm 1, consists of an initialization step, and of a recursive update of the estimates and their covariances. As already described, estimations are provided for the whole state vector $x_{k}$, including damage indexes $d_{k}$, and for the POMs in $\varphi_{l,k}$.

In the initialization step, the initial guess (in terms of ${\hat{x}}_{0}$ and ${\hat{\varphi }}_{l,0}$ and relevant covariance matrices) is first set, then all the filter particles x0j, with $j=1,\ldots ,N_{s}$ being the particle or sample index, are consistently selected along with the corresponding weights ω0j.

The state vector ${\hat{x}}_{0}$ can be easily determined according to initial conditions at time $t_{0}$. For instance, if the structural system is initially at rest, all the entries of vectors u and $\dot{u}$ are null, whereas entries of $\ddot{u}$ could be computed by solving Equation (1) at instant $t_{0}$; components of ${\hat{x}}_{0}$ can next be obtained once the POMs are initialized too. According to real situations encountered whenever a structural system (like a building) has to be monitored, we assume that at time $t_{0}$ a preliminary stage of training of the reduced-order model has been actually carried out. This means that, besides possible random fluctuations due to noises, the initial estimation of ${\hat{\varphi }}_{l,0}$ is indeed accurate. Vector ${\hat{\varphi }}_{l,k}$ will be subsequently updated to cope with a changing damage state leading to a variation in the structural responses to loading, avoiding the need of a time consuming retraining stage; for further details readers are referred to [14,19].

Within the subsequent time steps, particles are first drawn on the basis of the current expected value and covariance of the estimates, then allowed to (nonlinearly) evolve in time and, eventually, the estimations are updated by averaging what brought by the particles themselves. As proved in [39], the variance of the weights increases over time and, therefore, after few steps only one weight will be not negligible, while all the other ones will converge to zero. In order to avoid the computational costs associated with negligible terms, a resampling stage is adopted to select only the most likely particles; accordingly, uj is a random value drawn at this stage from the uniform probability distribution U[0,1]. Finally, the tracking of the POMs of the reduced-order model is obtained with Kalman filter; hence, no particles have been defined for this step of the proposed SHM strategy. A subtle difference between the approach discussed above and what typically reported in the literature is that the estimation of the augmented state and POMs is pursued separately. Linearity of POMs evolution and the related observation equation, then allows computing a closed-form solution to their estimation through a Kalman filter. When POMs are updated, the orthogonality condition among them is destroyed and so they must be re-orthogonolized right after.

**Algorithm 1.** Scheme of the proposed damage identification and state tracking method-Initialization at $t_{0}$: $${\hat{x}}_{0}={L_{0}}^{\text{T}}E\left[x_{0}\right]$$
$$P_{0}={L_{0}}^{\text{T}}E\left[\left(x_{0}-E\left[x_{0}\right]\right){\left(x_{0}-E\left[x_{0}\right]\right)}^{\text{T}}\right]L_{0}$$
x0j=x^0ω0j=p(y0|x0j)   j=1,…,Ns
$${\hat{\varphi }}_{l,0}=E\left[\varphi_{l,0}\right]$$
$$P_{0}^{ss}=E\left[\left(\varphi_{l,0}-{\hat{\varphi }}_{l,0}\right){\left(\varphi_{l,0}-{\hat{\varphi }}_{l,0}\right)}^{\text{T}}\right]$$-Recursive computation at $t_{k}=t_{1},\ldots ,T_{end}$; $j=1,\ldots ,N_{s}$Prediction stage:Draw samplesxk−j~ p(xk|xk−1j)Push particles toward the region of high probability through an EKF Pk−j=FkPk−1jFkT+W
Gkj=Pk−jHT(HPk−jHT+V)−1
xkj=xk−j+Gkj(yk−HTxk−j)
Pkj=Pk−j−GkjHPk−jUpdate stage:Evolve weights ωkj=ωk−1j p(yk|xkj)Resampling uj~U[0,1]
find m s.t. ∑i=1m−1ωki<uj<∑i=1mωki
xkj=xkm
ωk*j=1NsCompute expected value x^k=∑j=1Nsωk*jxkjPredict sub-space and associated covariance $\varphi_{l,k}^{-}={\hat{\varphi }}_{l,k-1}$
$P_{k}^{ss-}=P_{k-1}^{ss}+W^{ss}$Calculate Kalman filter gain for updating sub-space $G_{k}^{ss}=P_{k}^{ss-}H_{k}^{ss\text{T}}{\left(H_{k}^{ss}P_{k}^{i-}H_{k}^{ss\text{T}}+V\right)}^{-1}$Update sub-space and associated covariance ${\hat{\varphi }}_{l,k}=\varphi_{l,k}^{-}+G_{k}^{ss}\left(y_{k}-H_{k}^{ss}\varphi_{l,k}^{-}\right)$
$$P_{k}^{ss}=P_{k}^{ss-}-G_{k}^{ss}H_{k}^{ss}P_{k}^{ss-}$$

To investigate the computational aspects of incorporating a reduced-order model within the proposed hybrid particle-Kalman filtering scheme, the computational complexity of the procedure is considered in terms of the order of the required floating-point operations, and compared with that of the full order model. Concerning reduced-order modeling, the complexity associated with obtaining the reduced bases from the snapshot matrix is $O\left(n\;N_{snap}^{2}+l\;n\;N_{obs}+N_{snap}^{3}\right)$, where we recall that n is the number of DOFs of the full-order model, l is the number of POMs retained in the reduced-order one, and $N_{snap}$ stands for the number of snapshots collected, see [40]. Table 1 provides instead the computational complexity of all the stages of the proposed hybrid particle-Kalman filtering, as listed in Algorithm 1. In the prediction phase, the drawing of samples from a multivariate probability distribution scales linearly with the size $3l+N_{p}$ of the state vector, and the number $N_{s}$ of samples; next, by adopting an Extended Kalman Filter (EKF) to improve the quality of the particle ensemble, a square n × n matrix must be inverted and the related computational complexity is $O\left(n^{3}\right)$ according to a Gauss-Jordan procedure [41]. In the subsequent update stage, the complexity is dominated by the calculation of the determinant of the covariance matrix $P_{k}$ of the state vector; resampling is endowed with a $O\left(N_{s}\right)$ complexity, according to [42]. When dealing with the reduced-order modeling, an additional computational burden is associated to the updating of the sub-space, as reported at Stages 4–6 of the Algorithm. Overall, the computational complexity of the whole procedure is:

(11) $$O\left(n^{2}N_{obs}+N_{s}\left({\left(N_{p}+l\right)}^{3}+N_{obs}^{3}+N_{obs}{\left(N_{p}+l\right)}^{2}+N_{obs}^{2}\left(N_{p}+l\right)\right)\right)$$

In the case that the full-order model is used for hybrid particle-Kalman filtering, the relevant complexity instead reads:

(12) $$O\left(N_{s}\left({\left(N_{p}+n\right)}^{3}+N_{obs}^{3}+N_{obs}{\left(N_{p}+n\right)}^{2}+N_{obs}^{2}\left(N_{p}+n\right)\right)\right)$$

By making recourse to the above defined relations for complexities associated with the use of reduced- and full-order models in the hybrid particle filtering procedure, an estimate of the analysis speed-up can be computed as the ratio between the number of operations required to run the SHM with the full-order model and that required instead to run the SHM with the reduced-order model. It can be observed in Equations (11) and (12) that the complexity features at most a term $O\left(n^{2}\right)$ in the first case, and $O\left(n^{3}\right)$ in the second. The obtained complexities for the two procedures refer to asymptotic scenarios; the real CPU times and so the corresponding speed-up could be different from the estimates obtained via complexity due to the effects of, e.g., processor architecture, hardware settings, and structure of matrices.

**Table 1:** Computational complexity of the stages of the proposed algorithm.

		Computational Complexity (Flops)
prediction	1	$O\left(N_{s}\left(l+N_{p}\right)\right)$
	2	$O\left(N_{s}\left({\left(l+N_{p}\right)}^{3}+\;N_{obs}{\left(l+N_{p}\right)}^{2}+N_{obs}^{2}\left(l+N_{p}\right)+N_{obs}^{3}\right)\right)$
update	1	$O\left(N_{s}{\left(l+N_{p}\right)}^{3}\right)$
	2	$O\left(N_{s}\right)$
	3	$O\left(N_{s}\left(l+N_{p}\right)\right)$
	4	$O\left(n^{2}\right)$
	5	$O\left(n^{2}N_{obs}+n\;N_{obs}^{2}+N_{obs}^{3}\right)$
	6	$O\left(nN_{obs}+n\;N_{obs}^{2}\right)$

## 3. Results and Discussion

We consider in this Section a benchmark test represented by a thin plate subject to bending. We provide first the geometrical features of the plate and the mechanical properties of the material considered, along with loading/boundary conditions. We next discuss possible effects on the health monitoring capabilities of the major parameters of the proposed approach, namely: the number l of the POMs retained in the reduced-order model; the process noise; the measurement noise. We provide outcomes at varying damage level in one region of plate only, and also at a varying spreading of the damage over the plate. Finally, data are reported considering the speed-up provided by the reduced-order modeling, at varying space discretization adopted for the initial full-order finite element model.

**Figure 1:**
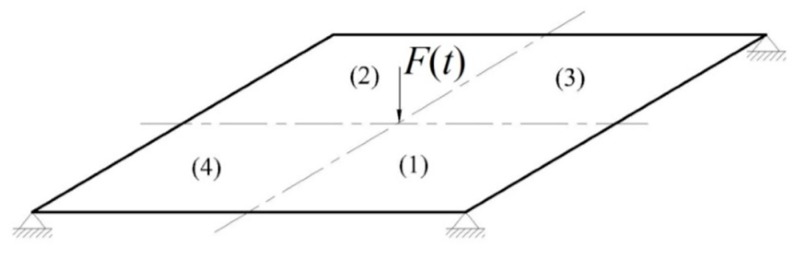
Benchmark plate test: boundary and loading conditions, and zone numbering.

As shown in Figure 1, the structure to be monitored is a thin square plate, with side length 200 mm and thickness 5 mm. The plate is assumed to be made of aluminum (6061-T6), whose relevant mechanical properties (in the virgin state) are: Young’s modulus E = 68.9 GPa, Poisson’s ratio ν=0.3, and density ρ=2500 kg/m^3^.

The plate has been modeled with the commercial finite element code Abaqus, using the S4R general-purpose, conventional shell elements which take into account also transverse shear deformations [43]. The kinematics of this element type is fully characterized by six DOFs per node, that are the three displacements along the axes of any orthonormal reference frame (typically with two of them belonging to the mid-plane of the plate and one pointing perpendicularly to such mid-plane) and the three rotations about the same axes.

The plate is simply supported at the four corners, and subject to a sinusoidally varying force $F\left(t\right)=F^{M}\text{sin}\left(2\pi ft\right)$ at its center (Figure 1). The maximum value of the force $F^{M}=100$ N has been set to avoid damage development in the plate caused by it; the plate is therefore considered already damaged during the test, and to possibly suffer damage growth due to external causes. The frequency of the load has been instead set to f=80 Hz, which is smaller than the fundamental frequency of vibrations of the aluminum plate, which amounts to $f^{\prime }=315$ Hz for the undamaged case, and $f^{\prime \prime }=280$ Hz for the reference damage case described in what follows.

The structure is decomposed into four regions, each of them associated with the relevant target damage index $d_{i}$, with i=1,…,4. If not stated otherwise, in the examples to follow the structure is supposed to be damaged only in zone 2 (see Figure 1), where the Young’s modulus is reduced to one half of its virgin value: therefore, $d_{2}=0.5$ is the target damage value to be estimated.

In a pseudo-experimental test frame, finite element analyses have been adopted to first obtain the mass matrix M of the whole system and the (undamaged) stiffness submatrices $K_{und}^{i}$ (see Equation (6)) of the mentioned $N_{p}=4$ regions. Next, for each damage scenario here considered, the same code has been used to run the full-order analysis (although it can be run independently, once the mass and stiffness matrices have been obtained); accordingly, the snapshots are collected for the considered loading/boundary conditions, and POMs in ${\hat{\varphi }}_{l,0}$ are obtained. As for measurements, results of the simulations have been corrupted with a Gaussian noise of known variance according to Equation (7). For the plate bending model considered in this paper, rotations about the in-plane reference axes, at the mid-point of each plate edge are handled as observations; this is in line with the optimal spatial distribution of measurements gathered through a network of surface-mounted inertial sensors, proposed in [28,29] to achieve sensitivity to damage independently of its location and amount.

The uncertainty and noise levels in the model are quantified for the structural system by the time-independent matrices W and V (Section 2.2). As for the process noise, which takes into account the uncertainties introduced by the model (due to the spatial and temporal discretization and to the order reduction procedure), its covariance W is scaled by a dimensionless factor $\sigma_{w}$, having the role of a standard deviation for the DOFs in the reduced-order model, according to:

$$W=\sigma_{w}^{2}I_{w}$$

where $I_{w}\in {\mathbb{R}}^{3l+N_{p}}$ is an identity matrix of appropriate size.

The measurement noise takes instead into account what related to the physical medium of communication between the sensors and the acquisition system, the random electrical noise due to the sensors and the electrical circuits, the quality of the measurement devices and also the environmental noise. Similar to the process noise, the measurement covariance V is scaled by a dimensionless factor $\sigma_{v}$, playing the role of the standard deviation of the collected rotation angles, according to:

$$V=\sigma_{v}^{2}I_{v}$$

where now $I_{v}\in {\mathbb{R}}^{N_{obs}}$. So, all the measurements are assumed uncorrelated. It is well-known that the performance of Bayesian filters rests on a proper choice of the above covariance matrices. By tuning the values of the process and measurement noise variances, the level of confidence in the model and measurements equations can be set; for instance, in case the measurements are deemed more reliable than the model, a low measurement noise variance $\sigma_{v}^{2}$ should be assigned, when compared with the process one $\sigma_{w}^{2}$. In practice, measurement noise variance is known for the used sensors; tuning of the process noise variance is in general more intricate, and is normally achieved by a trial and error procedures offline. The optimal estimation of noise parameters in an online fashion has recently gained attention; reader are referred to [36,37,44] for further details.

In this study, the number of samples in particle filtering has been always assumed $N_{s}=10$. Moreover, to build the initial POMs, a number of snapshots $N_{snap}=50$ always led to convergence of those retained in the analyses.

**Figure 2:**
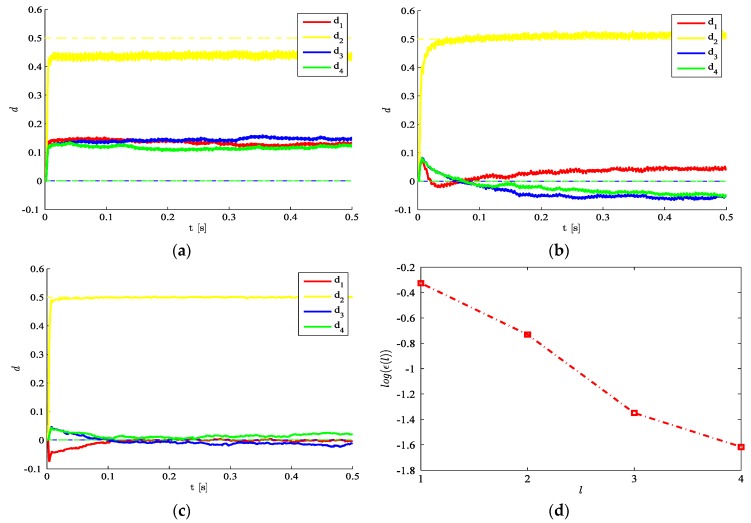
Time-invariant target damage state, $\sigma_{v}=10^{-5},\;\;\sigma_{w}=10^{-5},\;\;d_{0}=0$: time evolution of estimations of the damage indexes $d_{i}$, i=1,…,4 identified with (**a**) l=1; (**b**) l=2; (**c**) l=3; and (**d**) relative error $\epsilon \left(l\right)=\frac{{\left|\left|d_{l}-d\right|\right|}_{L^{2}}}{{\left|\left|d\right|\right|}_{L^{2}}}$ in the damage indexes, at varying order l of the reduced-order model.

Moving to the results, let us first investigate the effects of the number l of POMs taken in the reduced-order model, on the accuracy of the estimations obtained. In Figure 2, it is possible to see that such accuracy grows as the number of POMs gets higher. If the plate is coarsely meshed using only one finite element for each single region, the resulting structural DOFs turn out to amount to 50, once the constraints at plate corners are taken into account. Although both the geometry and the loading condition are two-fold symmetric, such symmetry has not been exploited in the analyses, as damage in only one region breaks it. A remarkable result reported in Figure 2d is the proof that, for this benchmark, it is possible to reduce the number of DOFs to only 4 and get an estimation error smaller than 10%.

**Figure 3:**
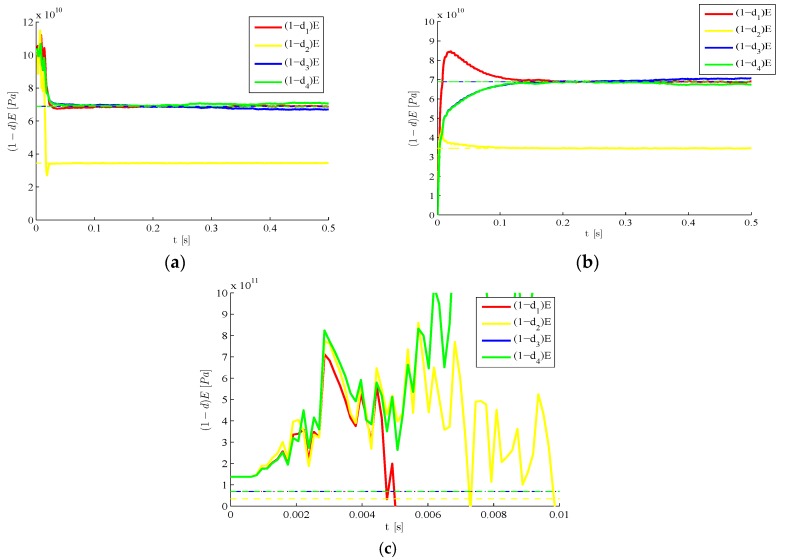
Time-invariant target damage state, $l=3,\;\;\sigma_{v}=10^{-5},\;\;\sigma_{w}=10^{-5}$: time evolution of estimations of the scaled elastic moduli (and, therefore, of the damage indexes $d_{i}$), identified with initial values (**a**) $E_{i,0}=\frac{3}{2}E$, i=1,…,4; (**b**) $E_{i,0}=0$; and (**c**) $E_{i,0}=2E$.

To assess the influence on the results of the initialization of the filter in terms of the damage state $d_{0}$, Figure 3 shows for l=3 that estimates are stable whenever the initial guess is close enough to the target one. Such performance test guarantees a wide range of applicability of the method, since the undamaged state $d_{0}=0$ and also an underestimation by 50% of the initial elastic moduli allow to assure stability. The other way around, an initial guess too far from the target solution (Figure 3c) is shown to insert instabilities or biases in the estimations.

**Figure 4:**
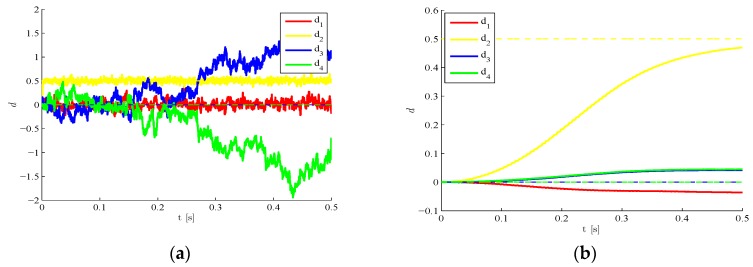
Time-invariant target damage state, $l=3,\;\;\sigma_{v}=10^{-5},\;\;d_{0}=0$: time evolution of estimations of the damage indexes $d_{i}$, identified with a process noise (**a**) $\sigma_{w}=10^{-2}$ and (**b**) ${\text{σ}}_{\text{w}}=10^{-5}$.

If we consider the effects of the process noise, some exemplary results are gathered in Figure 4. As expected, a high noise level amounting to $\sigma_{w}=10^{-2}$ (Figure 4a), can incept large variations of the estimates over each time interval, and therefore gives rise to possible biases or even divergence in the final solution. On the other hand, if the process noise is lowered to $\sigma_{w}=10^{-5}$ (Figure 4b), wild oscillations do not show up any longer and the estimation evolutions become smooth.

**Figure 5:**
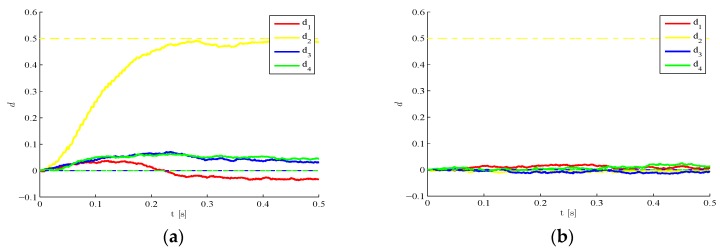
Time-invariant target damage state, $l=3,\;\;\sigma_{w}=10^{-5},\;\;d_{0}=0$: time evolution of estimations of the damage indexes $d_{i}$, identified with a measurement noise featuring (**a**) $\sigma_{v}=1.5\times 10^{-5}$ and (**b**) $\sigma_{v}=10^{-3}$.

In the analyses so far, the measurement noise level was kept low through $\sigma_{v}=10^{-5}$, *i.e*., (even if not explicitly shown) smaller than 10% of the maximum amplitude of the structural response under the considered loading. Figure 5 testifies that, as the measurement noise gets bigger (up to $\sigma_{v}=10^{-3}$), the ability of the procedure to estimate the damage indexes drops; as results are here shown for l=3, they can be compared to the plots already reported in Figure 2a for $\sigma_{v}=10^{-5}$. The trend shown by the accuracy of the estimates and by the readiness to approach the target values is basically due to the fact that the structural response gets hidden by the noise if $\sigma_{v}$ becomes bigger, and the filter is not able to extract significant information from measurements. Overall, by increasing $\sigma_{v}$ the transitory stage in the time evolution of the damage indexes lasts more and more (Figure 5a), or the SHM procedure can even lose the ability to estimate the damage in a region (Figure 5b). This response has been checked to vary monotonically and smoothly when $\sigma_{v}$ changes; moreover, plots in Figure 5 are reported for a null initialization of damage parameters, namely for $d_{0}=0$, but similar results can be obtained for any other initial guess guaranteeing the stability of estimations.

**Figure 6:**
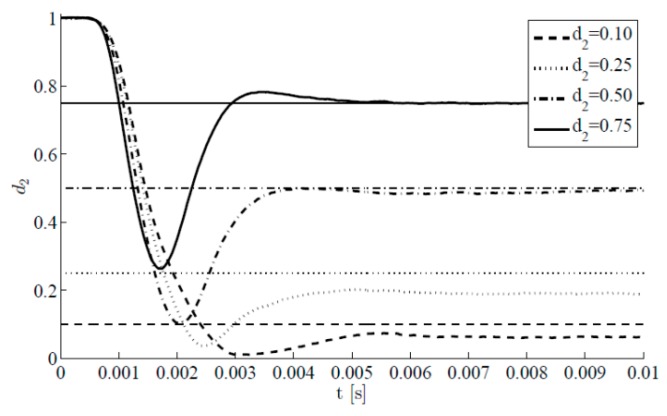
Time-invariant target damage state, $l=3,\;\;\sigma_{v}=10^{-5},\;\;\sigma_{w}=10^{-5},d_{0}=1$: time evolution of estimations of the damage index $d_{2}$ at varying target value.

We next investigate the method performance at a varying value of the target damage index $d_{2}$, with l=3 and still keeping the noise factors $\sigma_{v}=\sigma_{w}=10^{-5}$ adopted in Figure 3. Figure 6 shows the results in terms of time evolutions of the estimate of $d_{2}$ for the four target cases $d_{2}=0.1,\;0.25,\;0.5,\;0.75$. Such evolutions are obtained along with those relevant to the other damage indexes (all zero valued); since the trend shown by these latter ones is basically the same reported in Figure 3b, we focus on the initial transient stage of the estimations of $d_{2}$ starting from the initial guess $d_{2}=1$. Such transient stage is reported to last less than 0.01 s; after that, estimates are affected by small fluctuations only. It also appears that target values are promptly matched if damage values are large. This is somehow expected, as small target values of $d_{2}$ provide small drifts from the response of the healthy structure, and so the sensitivity to damage of the monitoring system in the noisy environment gets detrimentally affected.

**Figure 7:**
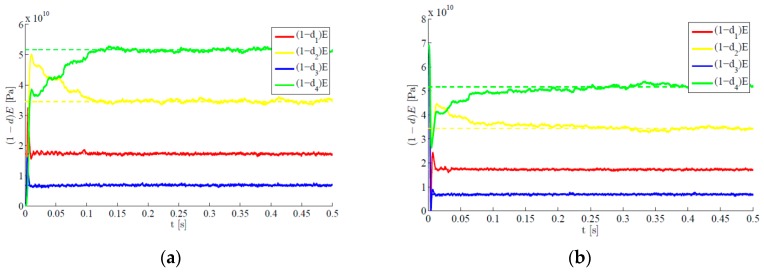
Time-invariant target damage state, four plate regions damaged, l=3  $\sigma_{v}=10^{-5},\;\;\sigma_{w}=10^{-5}$: time evolution of estimations of the scaled elastic moduli, identified with initial values (**a**) $d_{0}=1$ and (**b**) $d_{0}=0$.

The proposed methodology also features the same accuracy level if all the plate regions are damaged. Figure 7 provides the time evolutions of the estimates, in the exemplary case characterized by target values $d_{1}=0.75$, $d_{2}=0.5$, $d_{3}=0.9$ and $d_{4}=0.25$. For two different initialization sets, it is shown that estimates converge fast towards the target values, and small oscillations around them are next linked to the hybrid filtering methodology adopted. Comparing these results with those reported in Figure 6, it can be seen once again that smaller values of the damage indexes (and, so bigger values of the residual stiffness reported in the graphs) are connected to a delayed convergence of the filter estimates. Indeed, the provided steady-state solutions always perfectly match the target one.

**Figure 8:**
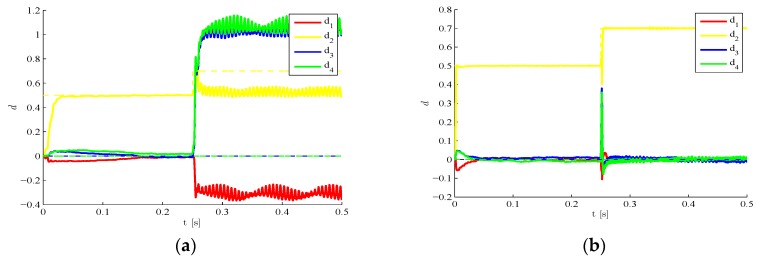
Time-varying target damage state, $l=3,\;\;\sigma_{v}=10^{-5},\;\;\sigma_{w}=10^{-5},d_{0}=0$: time evolution of estimations of the damage indexes $d_{i}$, identified (**a**) without update of POMs and (**b**) with POMs update.

We move now to a case characterized by a time evolving damage state. The structure is still assumed to be initially damaged with $d_{2}=0.5$, but such damage is suddenly increased to $d_{2}=0.7$ around t=0.25 s due to a further event, possibly linked in real-life situations to unexpected or extreme loadings. In Figure 8 results are provided concerning the estimated time evolution of the damage indexes, by either disregarding (Figure 8a) or allowing for (Figure 8b) the sub-space update. Such update, according to the setting defined in Section 2, is automatically driven by the filtering procedure as soon as the structural observations display a drift away from the response expected on the basis of the current damage state. POMs update is obviously compulsory to keep a similar degree of accuracy of the reduced-order model when the damage state is altered. If such update is not performed, the reduced-order model (that has been trained with the initial damage state) does not match the current structural health, and the damage estimates diverge or are affected by unacceptable biases.

**Figure 9:**
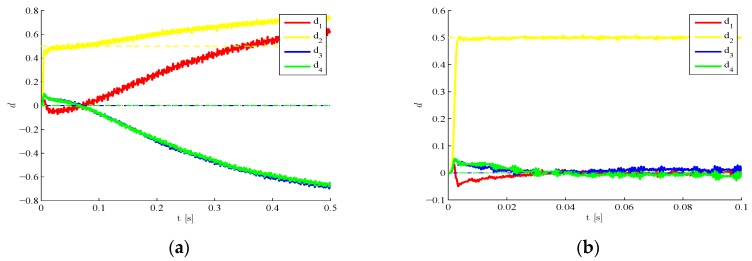
Time-invariant target damage state, fine space discretization, $\sigma_{v}=10^{-5},\;\;\sigma_{w}=10^{-5},\;\;d_{0}=0$: time evolution of estimations of the damage indexes $d_{i}$, identified with (**a**) l=1 and (**b**) l=2.

Some additional results are provided in Figure 9 in relation to a finer mesh adopted to build the full-order model; in this case, the free DOFs of the initial finite element model amount to 722. Compared to the former case linked to the coarsest possible mesh, a slightly higher number of POMs would be necessary to attain a high level of accuracy of the reduced-order model, if the proposed filtering procedure were not adopted. However, as stated in Section 2, the intricate formulation consisting in three filters processing the data, allows also to increase the accuracy of the reduced-order model, so that only two POMs prove enough to achieve an unbiased estimation of the damage state in a short time, see Figure 9b.

Results in the figures have shown that each damage index, although theoretically constrained to belong to the [0–1) interval, may be estimated out of the bounds on such interval. If damage becomes negative, then the stiffness properties of the structure results to be enhanced, hence greater than in the virgin state; that is obviously of no practical interest, and should be considered as a wrong outcome of filtering procedure. Anyhow, this kind of results does not violate the thermodynamic requirement to always have a positive reduced Young's modulus (see [34]). The other way around, damage can become greater than one, and so the reduced stiffness turns out to be negative. Although negative tangent stiffness values can be encountered at the structural level when local failure phenomena take place, in our case those values are to be considered wrong outcomes of the filtering procedure. Nonetheless, values of the damage indexes have not been constrained in our procedure, so that a standard particle-Kalman filter can be adopted. Alternatively, implementations centered on the so-called constrained Kalman filters were offered [45,46,47]; such possible alternative implementations have not been considered in our work since, as clearly depicted in the graphs of this Section, estimates of the damage indexes largely exceed the bounds only when the solution becomes unstable. In all the analyses providing reliable results, it usually happens that only some indexes slightly move below the zero value threshold.

Finally, concerning the analysis speed-up, relevant outcomes are reported in Table 2 at varying mesh and order l of the model, in terms of both CPU time and floating-point operation metrics. These data have been obtained by running the procedure implemented in Matlab (release 2014a) on a personal computer featuring an Intel Xeon E3-1270 V2 @ 3.50 GHz processor, with 8.00 Gb of RAM and Windows 7 64-bit as OS. It can be seen that the finer the full-order space discretization, the higher the speed-up; on the basis of a minimum reported speed-up value exceeding 30 for the coarse mesh, this joint use of intricate filtering schemes and reduced-order modeling can prove successful in the design of real-time SHM procedures.

Table 2 also shows that the speed-up computed through the algorithmic complexity discussed in Section 2.2, actually represents an upper bound on the real one given by the CPU time. It decreases at a smaller rate than that linked to CPU time when the number l of retained POMs is increased, but it converges to unitary values when l tends to the number n of total DOFs of the full-order model (actually it converges to values slightly less than one, as POM update is not carried out with the full-order model).

**Table 2:** Analysis speed-up provided by CPU time and flops, at varying mesh and order l of the reduced-order model.

l	Coarse Mesh		Fine Mesh	
	CPU Time	Flops	CPU Time	Flops
1	41.8	100	1319.5	1812
2	37.2	89	701.7	1810
3	33.3	79	611.7	1809

## 4. Conclusions

In this work, an online structural health monitoring procedure based on reduced-order modeling and recursive Bayesian filters has been proposed. Order reduction of the numerical model, used to track the structural behavior, has been achieved through proper orthogonal decomposition. Filtering has been centered around an extended Kalman-particle filter, whereas adaptivity of the reduced-order model has been obtained thanks to a further Kalman filter.

According to the results of a benchmark test on a thin square plate subject to bending deformations, the main outcomes shown here can be outlined as follows:

- the model order reduction method allows decreasing dramatically the number of system degrees-of-freedom, without losing accuracy as for the estimation of damage;
- The initial conditions for the damage state can be easily set, so that the estimation procedure keeps stability;
- Measurement and process noises should be small in comparison with the amplitude of measurements, to avoid biases in the estimates.

In future investigations, the proposed approach will be further extended to attack real-life, more complex cases in terms of structural geometry, material behavior and loading/boundary conditions. It is in fact well known that by increasing the number of parameters to be estimated, *i.e.*, by increasing the number of regions wherein damage is assumed to be homogeneous, problems connected to the curse of dimensionality arise. This generally means that by increasing the number of parameters to estimate, a lower accuracy of the filtering procedure is expected.

## Appendix A. Algorithmic Details

In this Appendix, we go through the main algorithmic details of the proposed procedure to handle the dual estimation of the continuously updated, reduced-order model of the structural system.

We start by showing how Equation (5) can be obtained from the equation of motion (Equation (2)) of the reduced-order model. Within the considered time step $\left[t_{k-1}\;\;t_{k}\right]$, the solution is advanced through the following three-stage scheme:

a. Predictor stage

$${\tilde{\alpha }}_{k}=\alpha_{k-1}+\Delta t{\dot{\alpha }}_{k-1}+\Delta t^{2}\left(\frac{1}{2}-\beta \right){\ddot{\alpha }}_{k-1}$$

$$\dot{{\tilde{\alpha }}_{k}}={\dot{\alpha }}_{k-1}+\Delta t\left(1-\gamma \right){\ddot{\alpha }}_{k-1}$$

b. Explicit integrator stage

$${\ddot{\alpha }}_{k}={ℳ}_{k-1}^{-1}\left({ℱ}_{k}-D_{k-1}\dot{{\tilde{\alpha }}_{k}}-K_{k-1}{\tilde{\alpha }}_{k}\right)$$

c. Corrector stage

$$\alpha_{k}={\tilde{\alpha }}_{k}+\Delta t^{2}\beta {\ddot{\alpha }}_{k}$$

$${\dot{\alpha }}_{k}=\dot{{\tilde{\alpha }}_{k}}+\;\Delta t\;\gamma {\ddot{\alpha }}_{k}$$

where: β and γ are the Newmark’s parameters (see [35]); and matrices ${ℳ}_{k-1}$, $D_{k-1}$ and $K_{k-1}$ are time-dependent, since the sub-space model can be continuously updated at the end of each time step.

To simplify the equations, we assume in what follows that system damping is null. It was already discussed in [23] that, whenever the system is continuously excited, the filter results will not be affected by the damping properties of the structure. By considering the components of the state vector $x_{k}$ related to the structural state only, arranged according to:

$$z_{k}=\left\{\begin{array}{c} \alpha_{k}\\ {\dot{\alpha }}_{k}\\ {\ddot{\alpha }}_{k} \end{array}\right\}$$

the relevant evolution equation can be written, as provided by the time integration scheme described above:

$$z_{k}=A_{k-1}z_{k-1}+b_{k-1}$$

where:

$$A_{k-1}=[\begin{array}{ccc} I-\beta \Delta t^{2}{ℳ}_{k-1}^{-1}K_{k-1} & \Delta tI-\beta \Delta t^{3}{ℳ}_{k-1}^{-1}K_{k-1} & \Delta t^{2}(\frac{1}{2}-\beta )[I-\beta \Delta t^{2}{ℳ}_{k-1}^{-1}K_{k-1}]\\ -\Delta t\gamma {ℳ}_{k-1}^{-1}K_{k-1} & I-\Delta t^{2}\gamma {ℳ}_{k-1}^{-1}K_{k-1} & (1-\gamma )dtI-\Delta t^{3}\gamma (\frac{1}{2}-\beta ){ℳ}_{k-1}^{-1}K_{k-1}\\ -{ℳ}_{k-1}^{-1}K_{k-1} & -\Delta t{ℳ}_{k-1}^{-1}K_{k-1} & -\Delta t^{2}(\frac{1}{2}-\beta ){ℳ}_{k-1}^{-1}K_{k-1} \end{array}]$$

$$b_{k-1}=\left\{\begin{array}{c} \Delta t^{2}\beta {ℳ}_{k-1}^{-1}{ℱ}_{k}\\ \Delta t\;\gamma {ℳ}_{k-1}^{-1}{ℱ}_{k}\\ {ℳ}_{k-1}^{-1}{ℱ}_{k} \end{array}\right\}$$

Entries of $A_{k-1}$ and $b_{k-1}$ vary in time not only because POMs are updated, but also because damage indexes $d_{k-1}$ affecting the stiffness matrix are tracked in their time evolution.

In a noisy environment, the whole process model thus becomes:

$$x_{k}=\left\{\begin{array}{c} z_{k}\\ d_{k} \end{array}\right\}=\left\{\begin{array}{c} A_{k-1}z_{k-1}+b_{k-1}\\ d_{k-1} \end{array}\right\}+\;w_{k}=f_{k}\left(x_{k-1}\right)+\;w_{k}$$

whose Jacobian matrix $F_{k}$ reads:

$$F_{k}=\left[\begin{array}{cc} A_{k-1} & \frac{\partial \left(A_{k-1}z_{k-1}\right)}{\partial d_{k-1}}\\ 0 & I_{d} \end{array}\right]\;\;\;$$

where $I_{d}\in {\mathbb{R}}^{N_{p}}$ is an identity matrix, and the upper-right term of the matrix is made of terms like:

$$\frac{\partial (A_{k-1}z_{k-1})}{\partial d_{k-1}^{i}}=[\begin{array}{ccc} \Delta t^{2}\beta {ℳ}_{k-1}^{-1}K_{und}^{i} & \Delta t^{3}\beta {ℳ}_{k-1}^{-1}K_{und}^{i} & \Delta t^{2}(\frac{1}{2}-\beta )\beta {ℳ}_{k-1}^{-1}K_{und}^{i}\\ \Delta t\gamma {ℳ}_{k-1}^{-1}K_{k-1} & \Delta t^{2}\gamma {ℳ}_{k-1}^{-1}K_{und}^{i} & \Delta t^{3}\gamma (\frac{1}{2}-\beta ){ℳ}_{k-1}^{-1}K_{und}^{i}\\ {ℳ}_{k-1}^{-1}K_{und}^{i} & \Delta t{ℳ}_{k-1}^{-1}K_{und}^{i} & \Delta t^{2}(\frac{1}{2}-\beta ){ℳ}_{k-1}^{-1}K_{und}^{i} \end{array}]$$

$K_{\text{und}}^{\text{i}}$ terms being already defined in Equation (6).
